# Multiplexing physical stimulation on single human induced pluripotent stem cell-derived cardiomyocytes for phenotype modulation

**DOI:** 10.1088/1758-5090/abce0a

**Published:** 2021-03-10

**Authors:** Worrapong Kit-Anan, Manuel M Mazo, Brian X Wang, Vincent Leonardo, Isaac J Pence, Sahana Gopal, Amy Gelmi, Anika Nagelkerke, Michele Becce, Ciro Chiappini, Sian E Harding, Cesare M Terracciano, Molly M Stevens

**Affiliations:** 1 Department of Materials, Imperial College London, London, United Kingdom; 2 Department of Bioengineering, Imperial College London, London, United Kingdom; 3 Institute of Biomedical Engineering, Imperial College London, London, United Kingdom; 4 National Heart and Lung Institute, Imperial College London, London, United Kingdom; 5 Current Address: Applied Chemistry and Environmental Science, School of Science, RMIT University, Melbourne, VIC 3001, Australia

**Keywords:** microfabrication, cardiomyocyte, physical interaction, cell shape, calcium handling, human induced pluripotent stem cells

## Abstract

Traditional *in vitro* bioengineering approaches whereby only individual biophysical cues are manipulated at any one time are highly inefficient, falling short when recapitulating the complexity of the cardiac environment. Multiple biophysical cues are present in the native myocardial niche and are essential during development, as well as in maintenance of adult cardiomyocyte (CM) phenotype in both health and disease. This study establishes a novel biofabrication workflow to study and manipulate hiPSC-CMs and to understand how these cells respond to a multiplexed biophysical environment, namely 3D shape and substrate stiffness, at a single cell level. Silicon masters were fabricated and developed to generate inverse patterns of the desired 3D shapes in bas relief, which then were used to mold the designed microwell arrays into a hydrogel. Polyacrylamide (PAAm) was modified with the incorporation of acrylic acid to provide a carboxylic group conjugation site for adhesion motifs, without compromising capacity to modulate stiffness. In this manner, two individual parameters can be finely tuned independently within the hydrogel: the shape of the 3D microwell and its stiffness. The design allows the platform to isolate single hiPSC-CMs to study solely biophysical cues in the absence of cell-cell physical interaction. Under physiologic-like physical conditions (3D shape resembling that of adult CM and 9.83 kPa substrate stiffness that mimics muscle stiffness), isolated single hiPSC-CMs exhibit increased Cx-43 density, cell membrane stiffness and calcium transient amplitude; co-expression of the subpopulation-related MYL2-MYL7 proteins; and higher anisotropism than cells in pathologic-like conditions (flat surface and 112 kPa substrate stiffness). This demonstrates that supplying a physiologic or pathologic microenvironment to an isolated single hiPSC-CM in the absence of any physical cell-to-cell communication in this biofabricated platform leads to a significantly different set of cellular features, thus presenting a differential phenotype. Importantly, this demonstrates the high plasticity of hiPSC-CMs even in isolation. The ability of multiple biophysical cues to significantly influence isolated single hiPSC-CM phenotype and functionality highlights the importance of fine-tuning such cues for specific applications. This has the potential to produce more fit-for-purpose hiPSC-CMs. Further understanding of human cardiac development is enabled by the robust, versatile and reproducible biofabrication techniques applied here. We envision that this system could be easily applied to other tissues and cell types where the influence of cellular shape and stiffness of the surrounding environment is hypothesized to play an important role in physiology.

## 1. Introduction

Myocardial infarction (MI) is the main cause of death worldwide, with surviving patients facing life-long medication use and possible progression toward heart failure [1]. MI causes unrepairable damage to cardiomyocytes (CMs) in the affected myocardium. Though CM regeneration occurs in the postnatal human heart, the capacity for renewal is physiologically insignificant and decreases with age [2]. The recent discovery of human induced pluripotent stem cells (hiPSCs) and their ability to differentiate into CMs initially boosted hopes among clinicians and patients [3, 4], as this might offer an attractive alternative to organ transplantation that could in principle supply an unlimited source of patient-specific CMs for personalized, regenerative medicine. However, full realization of the potential of hiPSC-CMs has been hindered by limited understanding of the molecular basis of hiPSC-CM development, making it impossible to apply the appropriate cues to optimize stem cell fate and phenotype [5].

Although much is known about the role of morphogens and transcription factors in CM specification [6], how the biophysical niche modulates their behaviors is still obscure. Tissue architecture during morphogenesis is controlled in part by biomechanical properties of the substrate (extracellular matrix) consisting of two main vital factors: 3D shape and physical tension. The phenotype of human adult CMs in physiologic settings is characterized as: an overall cylindrical geometry, a length-to-width ratio of ≈ 5–7 [7, 8], an unloaded sarcomere length of ≈ 1.8 *µ*m, an extensive network of t-tubules, and a large cell volume with a high density of mitochondria [9, 10]. It is this 3D shape that allows the human adult CM to adequately distribute and compartmentalize its ultrastructural domains [11], and exert contractility in the *µ*N range per cell. This behavior allows the heart to beat synchronously and generate enough force to pump blood throughout the body. Additionally, physical tension (i.e. matrix stiffness and geometrical constraints) can promote or demote the transition from an immature to an adult phenotype, demonstrating the importance of matrix stiffness and shape in guiding CM behavior [12, 13].

The ability to control tissue architecture by altering substrate stiffness and other physical cues enables researchers to avoid the phenotypical heterogeneity frequently encountered using conventional culture dishes or embryoid bodies for *in vitro* studies, while providing controllable parameters for directing niche features of hiPSC-CMs such as chemical and physical CM–CM communication. Cell fate directed by biomechanical cues has been dissected using biofabrication techniques to produce engineered matrices of tunable stiffness, for either physiologic or pathologic conditions. 2D rectangular patterns with an aspect ratio of 5–7 enhance the microstructure of CMs, as demonstrated both by increased contractility and aligned sarcomere structures [8, 14]. Recently, a 2D pattern of isolated hiPSC-CMs derived from patients was developed as an *in vitro* disease model, recapitulating diastolic dysfunction in hypertrophic cardiomyopathy at the 2D single-cell level [15]. Continuous surface grooves have been shown to effectively align CMs and increase anisotropic contractility, which better mimics the native myocardium [16]. Rodriguez *et al* studied the effect of stiffness, cell alignment and cell-cell contact on human embryonic stem cell-derived CMs in a 2D setting [17]. The study revealed the importance of substrate stiffness, cell alignment and cell–cell contact in CM maturation. Unfortunately, 3D environments meant to mimic physiological shape and surface features of adult CMs [18]are limited by fabrication challenges such as material bio-incompatibility [19].

Tunable hydrogels have been employed to model and study the effect of the extracellular stiffness in directing cell fate. It has been shown that CMs can sense the stiffness of their environment [20]. A time-dependent scaffold designed to increase its stiffness over time has been shown to improve CM differentiation [21], whilst an increase in substrate stiffness has been shown to promote twitch force and intracellular calcium in CM [22–24]. These fabricated platforms have proven the importance of single biophysical cues towards directing CM behavior. Stiffness and shape are important and must be considered when choosing biomaterials for downstream applications. Many biomaterials have been explored over the past decades, demonstrating their usefulness in cardiac *in vitro* applications, such as natural biomaterials (e.g. alginate [25], gelatin [26], collagen [27], albumin [28]) and synthetic ones (e.g. polydimethylsiloxane (PDMS) [29], PAAm [30, 31], polyacrylamide-co-acrylic acid (PAAm-*co*-PAAc) [32] or gold nanomaterials [33]). PAAm has been one of the most widely exploited hydrogels for studying mechanotransduction in CMs because it can be fabricated with wide range of stiffnesses, is easy to control over a large area, and is optically transparent [34, 35]. Incorporation of acrylic acid into PAAm hydrogels (PAAm-*co*-PAAc) ensures robust functionalization with proteins, eliminating unreliable UV exposure methods [36]. Although the effect of single physical cues has been widely explored [37, 38], the response of an individual hiPSC-CM to concomitant changes in niche shape and stiffness constraints in a 3D setting has not yet been demonstrated. A more thorough understanding of how these signals guide cells in health and disease is extremely relevant for the generation of hiPSC differentiation strategies that faithfully recapitulate the biology of mature tissues and organs, and uncover relevant information on the dynamic differences between physiologic and pathologic CMs. This concept requires engineering a new platform able to constrain CMs into relevant 3D shapes, whilst simultaneously physically isolating them from cell–cell contact within a stiffness-tunable environment.

Here, we develop a new platform capable of simultaneously providing multiplexed physical stimuli to isolated individual CMs while isolating physical cell**–**cell communication using soft lithography-based stiffness-tunable biomaterials. We additionally investigate the influence of our platform on gene expression, structure and calcium handling of isolated single hiPSC-CMs and thereby highlight the vital physical factors necessary to modulate the features of single hiPSC-CMs to serve the specific needs of cardiac regeneration.

## 2. Methods

### 2.1. Fabrication of silicon master

Hard master molds mimicking the shape of mature CMs in adult human myocardium were fabricated via UV photolithography. A silicon wafer (Boron p-doped, resistivity 1–30 Ohm cm, 4˝ diameter, University Wafers Inc) was oxygen-plasma cleaned at 300 W for 5 min. The cleaned wafer then underwent a transition bake at 95 °C for 10 min, prior to spin-coating photoresist SU-8 2010 (MicroChem Inc) at 1000 rpm for 30 s. This was followed by soft-baking, at 95 °C for 3 min. Patterns of aligned rectangles with dimensions of 70 *µ*m × 14 *µ*m, 40 *µ*m × 8 *µ*m, 98 *µ*m × 14 *µ*m and 56 *µ*m × 8 *µ*m (Length × Width; the depth of all micropatterns was 20 *µ*m) with gap distances of 20 *µ*m edge-to-edge were made on an acetate photomask (JD Photodata). The pattern was transferred using a MA6 mask aligner (Suss, Germany), which exposed the photoresist to UV light for a total of 70.8 s at a power of ∼5.6 mW cm^−2^. Post-exposure bake was then performed, at 95 °C for 4 min. The substrate was then immersed in SU-8 Developer solution (MicroChem Inc.) for 6 min, in order to develop the photoresist by dissolving the uncrosslinked photopolymer. Finally, the wafer was washed with abundant isopropyl alcohol to remove the SU-8 developer and dried under a gentle N_2_ stream. The micropatterned silicon master surfaces were then activated using oxygen plasma prior to gas phase deposition of trichloro(1 H,1 H,2 H,2 H-perfluorooctyl)silane (Sigma) to provide a hydrophobic surface, preventing polymer adhesion and allowing the reuse of the Si masters.

### 2.2. Fabrication of PAAm-co-PAAc substrate

The PAAm-*co*-PAAc hydrogel was synthesized using 40% (w v^−1^) acrylamide solution (BioRad), 2% (w v^−1^) *bis*-acrylamide solution (BioRad), and 0.4% (w v^−1^) acrylic acid (Sigma) in distilled water. In order to better control coupling of adhesion proteins to the stiffness-tunable hydrogel, PAAm-*co*-PAAc solution was synthesized by the addition of acrylic acid monomer into the mixture of acrylamide and *bis*-acrylamide, as previously published [36, 39]. For each substrate stiffness (560 Pa, 9.83 kPa, 44 kPa and 112 kPa), the final concentration of acrylamide and *bis*-acrylamide are followed 3/0.1, 7.5/0.3, 14/0.4 and 16/0.8 ratio, respectively. To stabilize the hydrogel from pH changes, 10% (v v^−1^) of 50 mM HEPES and 0.5% (v v^−1^) of 10 M NaOH were added. The chemicals were mixed gently using vortex. The mixture was degassed for 30 min in a vacuum chamber. Polymerization was initiated by 10% (w v^−1^) ammonium persulfate (Sigma) and 0.2% (v v^−1^) N, N, N′, N′-Tetramethylethylenediamine (Sigma). To mold the hydrogels, silicon sheets were cut into ring shapes with a thickness of 1 mm, the external diameter was cut to the size of a silicon master and the inner diameter was cut to create an area to accommodate the polymer mixture. The solutions were poured onto a fabricated silicon master, confined by the silicon ring. A transparent plastic sheet was used to enclose the poured solution. This plastic sheet made the thickness of hydrogel to 1 mm, the same size as the thickness of silicon sheet, before allowing polymerization in a humidified chamber at 37 °C for 30 min. The patterned hydrogel was washed extensively with Dulbecco’s phosphate-buffered saline (DPBS) and exposed to UV for 30 min for sterilization. Finally, the micropatterned hydrogel was crosslinked with Rat Tail Collagen I (Corning) using EDC and NHS (Sigma) [40].

### 2.3. Carboxylic group quantification

Carboxylic groups were quantified using 0.5 mM Toluidine Blue O (Sigma) in milli-Q water pH 10, adjusted using 10 M NaOH. The hydrogel was incubated with 0.5 mM Toluidine Blue O and washed three times with milli-Q water pH 10. The dye was subsequently desorbed using 50% (w v^−1^) acetic acid (Sigma) in mill-Q water. The supernatants were transferred to a transparent reading plate (Corning). The solutions were read at 630 nm absorbance to measure carboxylic acid concentration from a standard curve. (*N* = 4, *n* ⩾ 3, where *N* = independent measurements and *n* = number of technical replicates in each independent measurement).

### 2.4. Swelling ratio characterization

After fabrication of PAAm-*co*-PAAc hydrogel, the patterned substrate was imaged using a Nikon IX73 inverted microscope immediately after removal from a silicon master. The hydrogel was precut into an 8 mm circular hydrogel using a custom punch. The hydrogel was then incubated in RPMI 1640 supplemented with B27 (Thermo Fisher Scientific) for 7 d in a 37 °C humidified incubator, before taking the image again. The width and length ratio were measured using Fiji image analysis software (*N* = 3, *n* = 3).

### 2.5. 3D reconstruction of microwell

PAAm-*co*-PAAc solution was mixed with FluoSpheres (Nile Red)^®^ carboxylate-modified microspheres, 0.02 *µ*m, at 1:150 (v v^−1^). The hydrogel was fabricated according to section 2.2. The hydrogel was precut and imaged using a Leica SP5 inverted confocal microscope while immersed in DPBS. 3D image reconstruction was completed using Fiji image analysis software (*N* = 5, *n* ⩾ 3).

### 2.6. hiPSC culture and CM differentiation protocol

hiPSCs (Gibco Human Episomal iPSC Line, Gibco) were maintained with Essential 8 medium (Thermo Fisher Scientific) on matrigel coated six-well plates (Corning). Cells were passaged every 4 d at a 1:12–1:20 ratio, using 0.5 mM EDTA (Invitrogen) in DPBS (Invitrogen). The splitting ratio was adjusted in order to reach full confluency at day 4. The medium was changed daily. Cells were routinely checked for the expression of pluripotency markers (OCT4, SOX2, NANOG), mycoplasma and bacterial contaminations.

To produce human CMs from hiPSCs, cells were differentiated into hiPSC-CMs with an adapted chemically-defined protocol [4, 41]. Briefly, hiPSCs were first treated with a small molecule inhibitor of GSK3β, CHIR99021, in RPMI 1640 (Life Technologies) supplemented with B27 minus insulin (Thermo Fisher Scientific) to activate the Wnt signaling pathway. After 48 h, the medium was replaced with RPMI 1640 supplemented with B27 minus insulin. After 24 h, cells were supplemented with a Wnt signaling inhibitor, Wnt-C59 in RPMI 1640 supplemented with B27 minus insulin, until day 5. On day 7, cells were cultured in RPMI supplemented with B27, and medium was changed every 2 d. To purify the CMs, the cell population was glucose-starved and supplemented with 5 mM sodium D-lactate in RPMI 1640 and B27 for 2–4 d to metabolically select hiPSC-CMs. These hiPSC-CMs were maintained in RPMI 1640 supplemented with B27.

### 2.7. Single cell seeding

hiPSC-CMs were dissociated as follows: Briefly, the hiPSC-CMs were washed three times with 0.5 mM EDTA in DPBS. Then, TrypLE (Gibco) was added to the cells and incubated at 37 °C for 10 min before vigorously pipetting to remove cells from the culture plate. RPMI 1640 (Life Technologies) supplemented with B27 (Thermo Fisher Scientific) was added to the cell suspension to neutralize the protease in TrypLE. The cell suspension was centrifuged at 200 g for 10 min before being resuspended in RPMI 1640supplemented with B27, 10%v v^−1^ fetal bovine serum (Gibco) and 10 *µ*M Y27632 (Biorbyt). 1 ml of this solution was added per 1 million cells. The cells were then disaggregated using a 21 G needle and passed through a 100 *µ*m cell strainer (Falcon). In parallel, the patterned hydrogel was precut into 11 mm diameter circles and placed into a 48 well plate; excess liquid was removed using a sterile gauze. Single cells were seeded to the hydrogel at a density of 50 000 cells per cm^2^ before removing excess solution.

### 2.8. Atomic force microscopy (AFM)

Contact mode AFM (Agilent) was used with Nanosensors AFM Probe qp-BioAC (Windsor Scientific) and analyzed using the Hertz model for 200 nm indentation with AtomicJ [42]. In the Hertz model, contact of non-adhesive and continuous surfaces, where minimal tension force exists, is assumed. Cells were cultured on the 3D micropatterned substrate for 7 d before the measurements. Medium was replaced with phenol red-free RPMI 1640 (Life Technologies) supplemented with B27 (Thermo Fisher Scientific) 24 h before measurement. A CMOS camera was used to visualize and identify single isolated hiPSC-CMs. During the measurement, a heated stage maintained the temperature at 37 °C (*N* = 4, *n* ⩾ 4).

### 2.9. Immunofluorescent staining

Samples were fixed with 4% PFA (Sigma) at room temperature for 30 min, washed three times with DPBS, permeated with 0.2% v v^−1^ Triton X-100 (Sigma Aldrich) in DPBS for 15 min, washed with DPBS and incubated with 10% (v v^−1^) donkey serum (VWR International), 5% (w v^−1^) bovine serum albumin (Sigma Aldrich), and 0.3 M glycine (Sigma Aldrich) for 1 h. A combination of two antibodies and dilutions from the following were chosen to characterize the morphology of single isolated hiPSC-CMs: anti-*α*-actinin, 1:200 (EA-53, monoclonal, Sigma); anti-cardiac troponin T (cTnT), 1:200 (Abcam); anti-Connexin-40, 1:200 (Abcam); anti-Connexin-43, 1:100 (Abcam); anti-Connexin-45, 1:500 (Abcam); anti-MYL2, 1:140 (Abcam); and anti-MYL7 1:400 (Abcam) overnight at 4 °C in DPBS. Afterward they were washed extensively with DPBS before incubating at a 1:400 dilution with two appropriate secondary antibodies(donkey anti-mouse immunoglobulin G, donkey anti-rabbit or donkey anti-goat (all IgG, H + L, Thermo Fisher Scientific)) in DPBS for 1 h. Nuclei were stained with 1:500 DAPI (Thermo Fisher Scientific) in DPBS for 1 min. The samples were mounted with Fluoromount Aqueous Mounting Medium (Sigma Aldrich) and imaged with a Zeiss LSM-780 confocal microscope. Cx-43 surface area analysis were performed using Fiji image analysis software (*N* ⩾ 5, *n* ⩾ 3).

### 2.10. Sarcomere length quantification

The samples were stained with anti-*α*-actinin and imaged as described in section 2.9. The images were processed with Fiji image analysis software. A line was drawn perpendicularly to several consecutive sarcomeres, resulting in a fluorescence intensity profile where peaks corresponded to the consecutive sarcomeres (*Z*-lines). The length of the line was then divided by the number of peaks. Three images per slice were acquired and the sarcomere length of at least five cells per image was measured (*N* = 3, *n* ⩾ 3).

### 2.11. Single cell quantitative polymerase chain reaction

Single cells were collected using a single use glass pipette of a Scanning Ion Conductance Microscope. Negative pressure was applied to create a small suction to remove a single isolated hiPSC-CM from the platform. The sample was then collected in a low protein binding tube (Eppendorf). Total RNA was isolated using the Single Cell Lysis Kit (Thermo Fisher Scientific), according to the manufacturer’s protocol, and cDNA produced using SuperScript^®^ VILO™ cDNA Synthesis Kit (Thermo Fisher Scientific), assuming a 1:1 conversion. cDNA was pre-amplified with 0.2X Taqman^®^ probes, see below, and TaqMan^®^ PreAmp Master Mix (Thermo Fisher Scientific) and diluted with TE buffer to 133 *µ*l. Single cell Quantitative PCR was performed using 4 *µ*l of cDNA with TaqMan^®^ Fast Advanced Master Mix (Thermo Fisher Scientific) and QuantStudio™ 6 Flex Real-Time PCR (Thermo Fisher Scientific) and the following TaqMan^®^ probes: GAPDH (Hs02758991_g1), MYL2 (Hs00166405_m1), MYL7 (Hs01085598_g1), MYH6 (Hs01101425_m1), MYH7 (Hs01110632_m1), TNNI1 (Hs00913333_m1) and TNNI3 (Hs00165957_m1). The ΔΔCt method was used to compare expression between single hiPSC-CMs cultured on 3D micropatterned substrates and flat control surfaces and normalized to hiPSC-CMs before plating (*N* = 4, *n* ⩾ 10).

### 2.12. Live Ca^2+^ imaging

GCamP6f was kindly provided by Professor Bruce Conklin (Gladstone Institutes) and differentiated as outlined in section 2.6 and recorded in Tyrode’s solution. To create this solution, the following were added to 1 l of dH_2_O while mixing using a magnetic stirrer: 8.18 g of NaCl (140 mM), 0.3375 g of KCl (6 mM), 1.86 g of glucose (10 mM), 2.38 g of HEPES (10 mM), 1 ml of 1 M MgCl_2_ solution (1 mM) and 1.8 ml of 1 M CaCl_2_ solution (1.8 mM) and adjusted with 2 M NaOH to pH 7.4. The samples were kept under superfusion of 37 °C Tyrode’s solution. The cells were stimulated at 1 Hz using MyoPacer (IonOptix) The records were obtained using a Nikon IX73 Inverted Microscope equipped with a Hamamatsu Flash 4 Orca and LED illumination (Cairn Research) by WinFluor software (University of Strathclyde). Raw data from WinFluor were preprocessed using Fiji image analysis software to obtain a fluorescent intensity profile. The data were analyzed by a blinded investigator. The records were analyzed using a custom MATLAB^®^ code (*N* = 4, *n* ⩾ 7).

### 2.13. Flow cytometry

Dissociated hiPSC-CMs were transferred to cytometry tubes and washed with DPBS. Fixable live/dead staining was used to remove dead cells from the analysis. FluoroFix buffer (BioLegend) was used before using Fix and Perm Cell Permeabilization Kit (Thermo Fisher Scientific). Anti-Troponin T Cardiac Isoform (clone 13–11; Thermo Fisher Scientific) diluted 1:500 was used to label cTnT and followed by secondary staining with Alexa Fluor^®^ 647 donkey anti-mouse IgG (H + L) (Life Technologies) diluted 1:500. The samples were kept overnight at 4 °C before analysis (BD Fortessa) (*N* = 3, *n* = 3).

### 2.14. Statistical analysis

Gene expression statistical analysis was performed in MATLAB and R. Normalized relative fold change values for each gene of interest were incorporated individually and as a ratio for the combinations of 3D topography and stiffness. Principal component analysis (PCA) was performed using PLS toolbox (Version 8.2.1, Eigenvector Research Inc.) for data reduction and visualization. Variable correlation was performed in MATLAB (R2015a, The Mathworks) using a Spearman’s correlation (‘corr’ function). For feature selection and sample classification, the PCR data was imported into R (version 3.4.0) and incorporated into the analysis as both normalized relative fold change and the indicated ratio combinations. Each group (3D topography-stiffness combination) was split into training and validation groups for a five-fold cross-validation of optimal model sparsity based on LASSO (*α* = 1.0) as implemented in the GLMnet package for multinomial regression. Following optimal parameter selection for winner features, the predicted probability for class membership was calculated for each sample based on a leave-one-sample-out cross-validation procedure. Predicted class membership was assigned for each sample to the class with the highest predicted probability. A resulting confusion matrix was generated based on known and predicted class membership. For the remainder of the data, statistical comparison of normally distributed data between groups was performed using a student-unpaired *t*-test, one-way or two-way analysis of variance (ANOVA) followed by Bonferroni’s test. These tests were performed using Prism 8 software (GraphPad). *P* values * < 0.05, ** < 0.01, and *** < 0.001 were considered statistically significant.

## 3. Results

### 3.1. Fabrication of the PAAm-co-PAAc hydrogel

**Figure 1.**
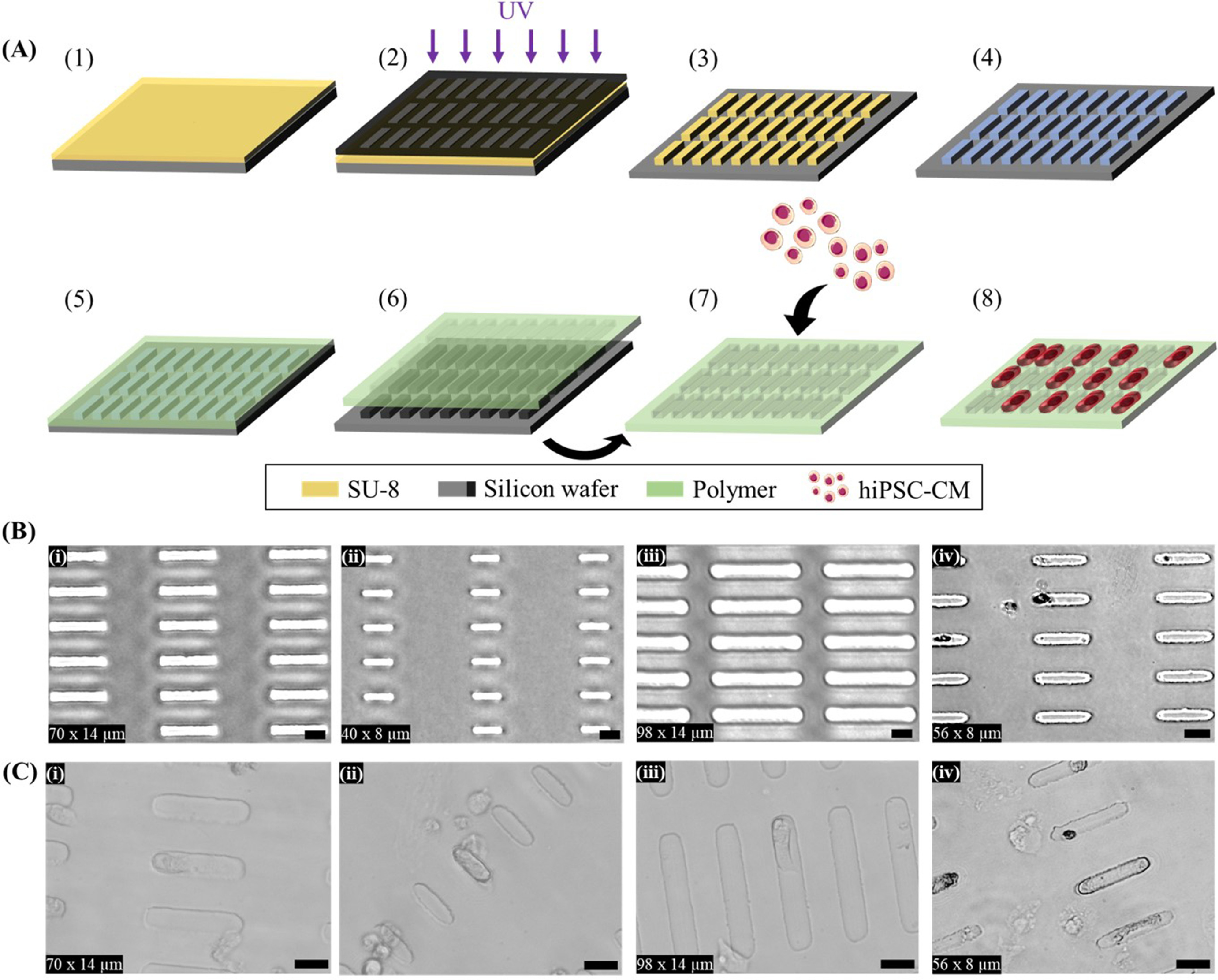
Microfabrication of polyacrylamide-*co*-acrylic acid (PAAm-*co*-PAAc) hydrogel and cell seeding. (A) Schematic representation of the microfabrication and cell-seeding workflow [1]: spin coating of photoresist (SU-8) [2], pattern transfer from photomask to photoresist by UV [3], pattern resulting from photolithography: arrays of pillars resembling the shape of adult CM [4], perfluorosilanization of substrate [5], PAAm-*co*-PAAc coating of the hard mold by drop-casting [6], removal of PAAm-*co*-PAAc and ECM conjugation [7], hiPSC-CMs seeding on the platform [8], hiPSC-CM culture on micropatterned PAAm-*co*-PAAc hydrogel. (B) Representative brightfield images of PAAm-*co*-PAAc hydrogels of (i) 70 × 14 *µ*m, (ii) 40 × 8 *µ*m, (iii) 98 × 14 *µ*m and (iv) 56 × 8 *µ*m. (C) Representative brightfield images of hiPSC-CMs cultured on (i) 70 × 14 *µ*m, (ii) 40 × 8 *µ*m, (iii) 98 × 14 *µ*m and (iv) 56 × 8 *µ*m. Scale bars: 25 *µ*m.

Firstly, we fabricated the multi array platform to function as a scaffold capable of simultaneously providing multiple biophysical cues in order to guide the development of single, physically isolated hiPSC-CMs *in vitro* (figure 1(A)). Using photolithography, we created an array of 3D microwells of the physiologic dimensions of adult human CMs, reported at an aspect ratio between 1:5 and 1:7 (width:length) [7, 43–45]. Figure 1(B) shows representative bright-field images of the 3D micropatterns on PAAm-*co*-PAAc hydrogel. This was achieved using different Si masters, fabricated to the different size of 3D micropattern. Further, the hydrogel platform can be fabricated to have several different patterns in a single hydrogel substrate by using another pre-designed Si master, thus making it perfectly possible to have multiple sizes or shapes within the same sample. Masters were developed to generate pillars, which molded the hydrogel to the shape of the designed microwell. This demonstrated the capability of the platform to tune 3D microfeatures for single cell manipulation. In order to mimic the collagen-rich extracellular matrix (ECM) found in the myocardium, known to play an essential role in cardiac remodeling [46], our platform was cross-linked via EDC-NHS coupling to collagen I. Figure 1(C) shows hiPSC-CMs seeded on the platform. The brightfield images showed that isolated single hiPSC-CMs can fully adapt to the 40 × 8 *µ*m of the 3D micropatterns. Therefore, we selected rectangular well (W) dimensions of 8 × 40 × 20 *µ*m for width, length, and depth, respectively, versus a flat control surface (F), each on physiologic (9.83 kPa, 7.5%/0.3%) and pathologic stiffness (112 kPa, 16%,0.8%) for the remainder of the study. Cell seeding density on the pattern also led to the highest number of isolated single hiPSC-CMs on a flat control surface (F).

### 3.2. PAAm-co-PAAc hydrogel characterizations

**Figure 2.**
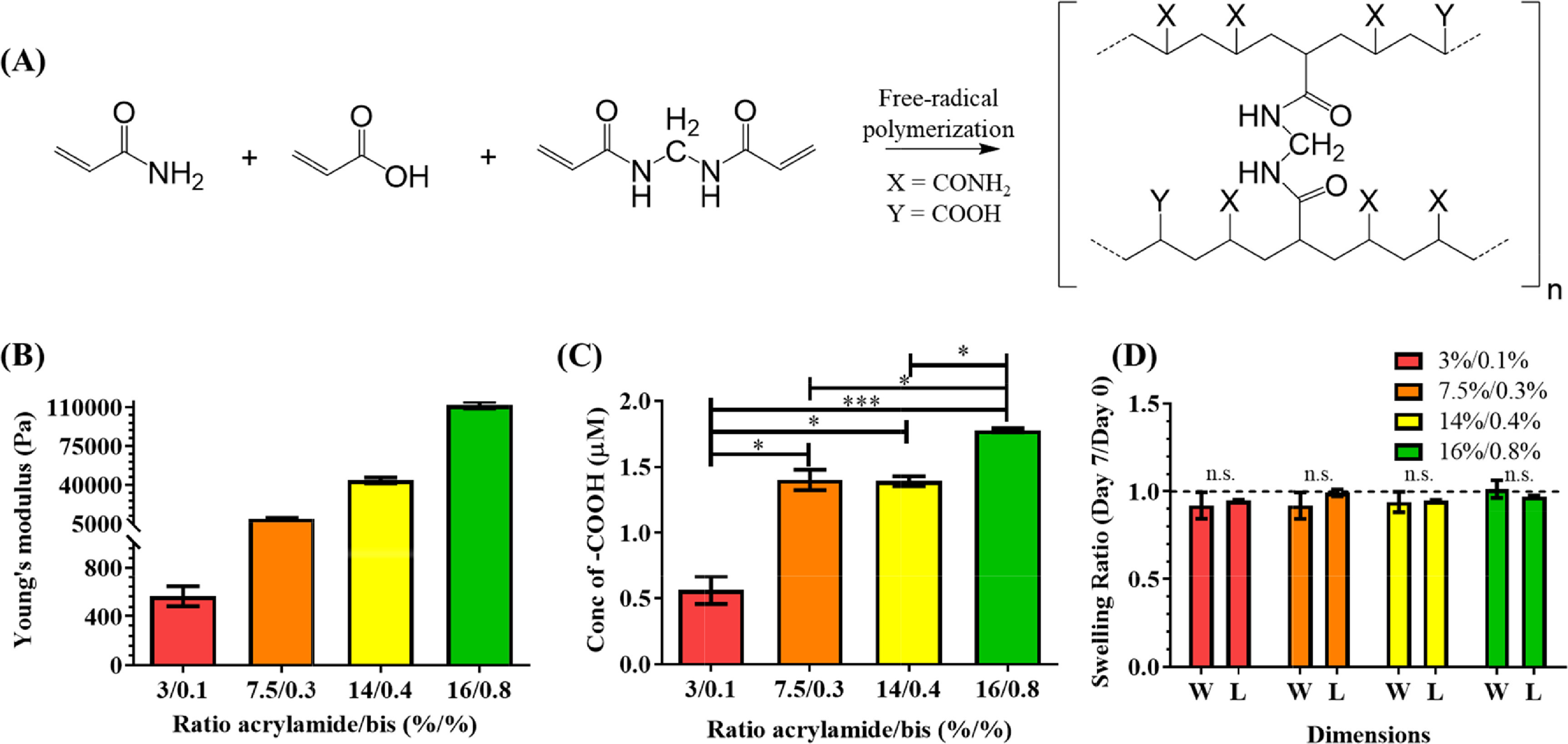
PAAm-*co*-PAAc hydrogel characterizations. (A) Chemical structure of PAAm-*co*-PAAc. (B) Young’s modulus measurement by contact-mode AFM. (C) Available carboxylic groups measured using Toluidine Blue O. (ANOVA, *p < 0.05, ***p < 0.001). (D) Swelling ratio of the 3D microwells showing no significant effect on the width (W) and length (L) directions after 7 d incubation in cell culture media (Student T-test, no significant differences). Dashed line represents no change (Swelling ratio = 1). Data shown as mean ± SEM. *N* = 3, independent experiments.

Next, we characterized the mechanical properties of PAAm-*co*-PAAc hydrogels (Chemical structure—figure 2(A)) using contact-mode AFM. As reported previously, varying the ratio of acrylamide and *bis*-acrylamide allows tailoring of substrate stiffness to that of physiologic (10–20 kPa) or pathologic (>50 kPa) myocardium [19, 47]. PAAm-*co*-PAAc hydrogels demonstrated capacity to form a range of stiffnesses between 560 Pa and 112 kPa (figure 2(B)). This demonstrated the capability of the platform to present a tunable stiffness, and also enabled matching the physiologic and pathologic range found in myocardium. The amount of carboxylic groups, from acrylic acid, was quantified using a Toluidine Blue O assay, as shown in figure 2(C), the 3%/0.1% mixture displayed the lowest concentration (0.56 *µ*M) while 7.5%/0.3% (1.40 *µ*M) and 14%/0.4% (1.39 *µ*M) showed a similar concentration of carboxylic groups. The 16%/0.8% mixture demonstrated significantly higher concentration (1.78 *µ*M) compared to the three aforementioned mixtures. This formulation (coupling to PAAm-*co*-PAAC via EDC-NHS) was able to conjugate a significantly increased amount of protein, as compared with the traditional Sulfo-SANPAH-unmodified PAAm (figure S1(A) (available online at stacks.iop.org/BF/13/025004/mmedia)), thus supporting our chosen strategy as an improved route to provide adhesion cues to hiPSC-CMs. The incorporation of acrylic acid enables better functionalization of proteins supporting cell adhesion, with protein density dependent on acrylic acid concentration, but also makes the hydrogel pH responsive by swelling and shrinking depending on the pH of the solution. Therefore, in order to prevent significant changes in the fabricated features, HEPES buffer was added to prevent pH sensitivity-related swelling and shrinking. To measure the swelling of the hydrogel, the width and length of the 3D microwells were measured after a 7 day incubation in the cell culture medium and compared to day 0 (immediately after polymerization). As shown in figure 2(D), no significant change in dimensions was found for any of the recipes. To further characterize the microwells, PAAm-*co*-PAAc hydrogel solution was mixed with Nile Red FluoSpheres^®^ to visualize the 3D microwells using z-stack images with a confocal microscope. The reconstructed images showed a 3D rectangular box as expected (Movie S1). To assess potential interference of the hydrophobic coating on the metabolic activity of the CMs, we measured CM metabolic activity on physiologic and pathologic stiffnesses. We found no significant difference in metabolic activity of hiPSC-CMs between the two different stiffnesses on PAAm-*co*-PAAc surface (figure S1(B)). Collectively, we have microfabricated an improved platform that can finely control multiple biophysical cues (i.e. 3D micropattern and substrate stiffness).

### 3.3. Physical multiplexed stimulation alters single hiPSC-CM’s cellular structures

**Figure 3.**
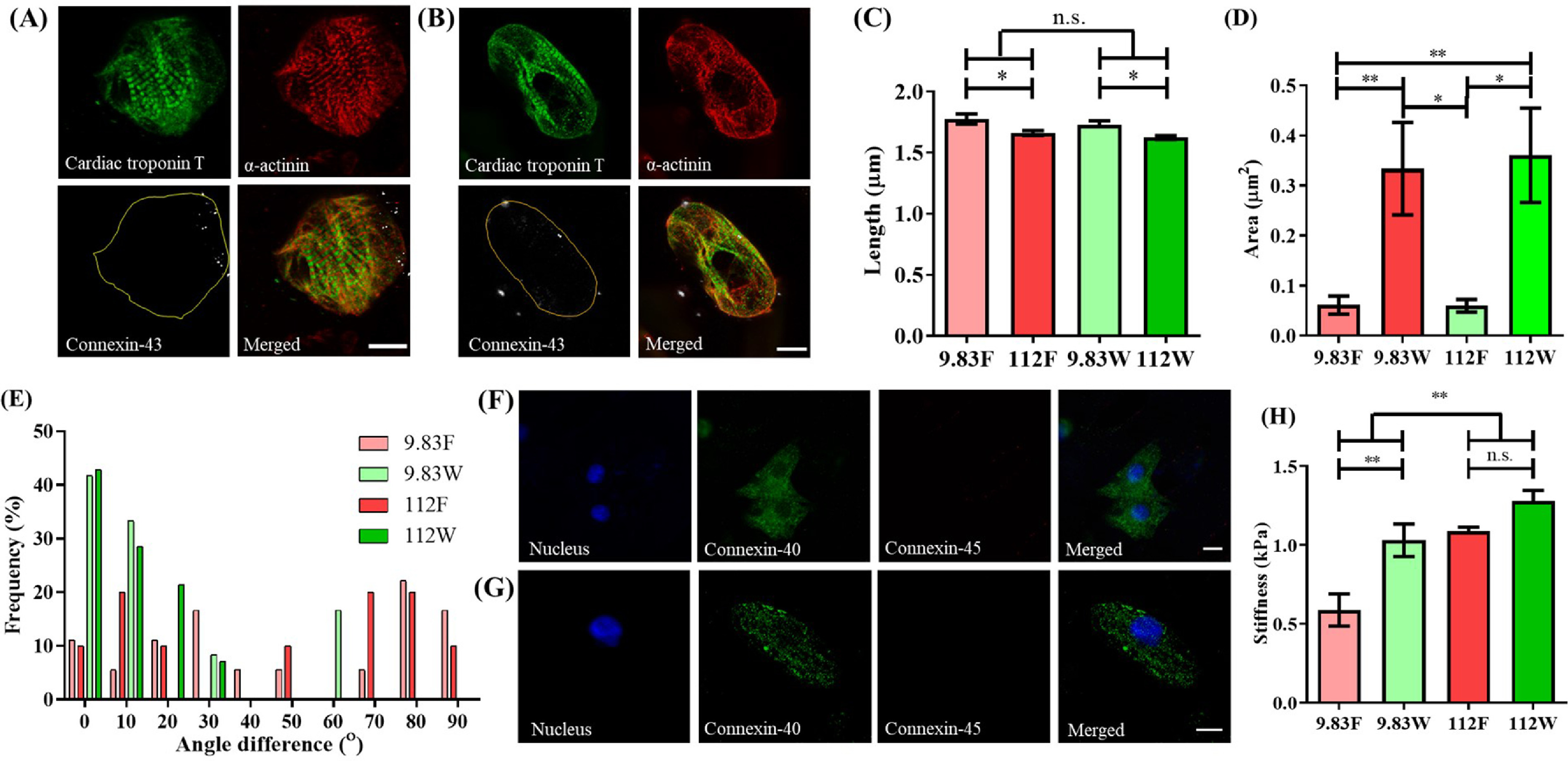
Concomitant modulation of cell shape and stiffness by PAAm-*co*-PAAc hydrogel influences the structure of isolated single hiPSC-CMs. Representative images of hiPSC-CMs stained for cTnT, *α*-actinin and Cx-43 on (A) the flat control surface and (B) inside the 3D adult-like microwell of physiologic substrate stiffness (9.83 kPa). Yellow outline demonstrates the perimeter of cell. (C) Sarcomere length showed a significantly higher spacing when cultured on physiologic stiffness than on pathologic surfaces (112 kPa) (2-way ANOVA, *p < 0.05). (D) Quantification of area occupied by Cx-43 on 3D microwells and flat surface controls, showing increased presence of Cx-43 in cells residing inside 3D microwells (2-way ANOVA, *p< 0.05, **p < 0.01). (E) Analysis of CM sarcomeric directionality, measured against *X*-axis, demonstrates capacity of the 3D microwells for inducing cell alignment. Representative images of Cx-40 and Cx-45 staining on (F) the flat control surface and (G) 3D microwell of physiologic stiffness. (H) Cell membrane stiffness measured using AFM, demonstrating increased stiffness under pathologic condition versus physiologic condition, as well as increased membrane rigidity of isolated single hiPSC-CMs residing inside the 3D microwells (2-way ANOVA, **p < 0.01). Scale bars: 10 *µ*m. Data shown as mean ± SEM, *N* ⩾ 3. (9.83 F = physiologic stiffness flat control, 9.83 W = physiologic stiffness 3D microwell, 112 F = pathologic stiffness flat control, 112 W = pathologic stiffness 3D microwell).

The myocardium is a functional syncytium, each CM being chemically and electrically connected to its neighbors by intercalated disks and connexins. However, to understand the molecular basis of how hiPSC-CMs respond to stiffness and 3D confinement, it is necessary to physically isolate them. So far, a quantitative and reliable method to track isolated single hiPSC-CMs is lacking, as many potential cardiac markers of the adult mature CMs revert to a fetal state under pathologic conditions [48–50]. To determine the capacity of our newly developed platform to perform quantitative and robust tracking of isolated single hiPSC-CMs, we employed multifactorial characterization of structural, genetic, and functional changes of isolated single hiPSC-CMs cultured on our platform. hiPSCs were differentiated to CMs, displaying a downregulation of pluripotency markers and a correct stepwise activation of cardiac genes, with a differentiation efficiency >85% as measured by FACS and immunofluorescent staining (figure S2). hiPSC-CMs were then isolated and plated on the platform. Isolated single hiPSC-CMs residing inside 3D microwells (movie S2A) and on flat surfaces (movie S2B) of the platform served as the treatment and control conditions, respectively. To evaluate the effect of the physical multiplexed platform on these isolated single hiPSC-CMs, the cells were cultured for 7 d on either physiologic or pathologic substrate stiffness. hiPSC-CM structural characterization was conducted to observe key CM features, including sarcomeres (via cTnT and sarcomeric-*α*-actinin distribution and localization) and gap junctions (via connexins 40, 43 and 45 (Cx-40, Cx-43 and Cx-45) distribution and localization). Sarcomere organization is directly related to function, with regularly spaced structures signaling an adult-like performance [8, 51]. Connexins on the other hand play a vital role in physical cell-cell communication, as well as being associated with cell phenotype: Cx-45 has dominantly been detected in the conductive tissue, whilst Cx-40 and/or Cx-43 are expressed in adult working myocardium (atrial and ventricular CMs), with a reduced expression (number and location) of connexins reported in MI and heart failure [52]. Staining for cTnT, sarcomeric-*α*-actinin, Cx-43, plus DAPI showed that isolated single hiPSC-CMs on flat controls (figure 3(A)) had higher anisotropy (random shape) compared to the cells residing in the 3D microwells (figure 3(B)), where they adapted to the predesigned 3D microwells. Furthermore, Cx-43 was distributed throughout the cell and its membrane on flat surfaces, while Cx-43 was localized near the 3D microwell’s wall. This demonstrated the capacity of the platform to affect connexin subcellular localization. Cells on substrates with physiologic stiffness (9.83 kPa) more closely resembled adult unloaded CMs, with Z-line spacings of 1.77 ± 0.12 *µ*m (F), 1.73 ± 0.11 *µ*m (W), compared to 1.66 ± 0.07 *µ*m (F) *µ*m and 1.62 ± 0.05 *µ*m (W), for those cultured on substrates with pathologic stiffness (112 kPa), independent of the presence of the 3D confinement (figure 3(C)) [53]. Additionally, the area of Cx-43 in isolated single hiPSC-CMs inside the 3D microwells was significantly greater compared to flat control in both substrate stiffnesses (figure 3(D)). A significant increase in sarcomere alignment was observed in cells residing in the adult-like 3D microwells, with isolated single hiPSC-CMs expressing their contractile structures following the long axis of the patterns, in contrast to isolated single hiPSC-CMs on flat control surfaces (figure 3(E)), thus demonstrating higher isotropy of single hiPSC-CMs cultured on the flat control surfaces. As shown in figures 3(F)**–**(G), isolated single hiPSC-CMs cultured in both conditions strongly expressed Cx-40, whereas Cx-45 was absent, pointing toward a working myocardium-like connexin expression. Finally, cell membrane stiffness was obtained from force-distance curves of the stress relaxation tests performed with AFM. Results demonstrated a significant increase in cellular stiffness (Young’s modulus) when isolated single hiPSC-CMs resided in 3D microwells in 9.83 kPa compared to a flat control surface on the same stiffness. (figure 3(H)). This demonstrated a development of membrane stiffness towards adult CM characteristics [21] in microwells. Together, cellular structure in the microwell was characterized by a more physiologic spacing and directional alignment of sarcomeres, a working CM-like connexin presence, more adult-like membrane stiffness, and an increased presence of Cx43 in spite of cell isolation.

### 3.4. Modulation of single cell gene expression in hiPSC-CMs by the platform

**Figure 4.**
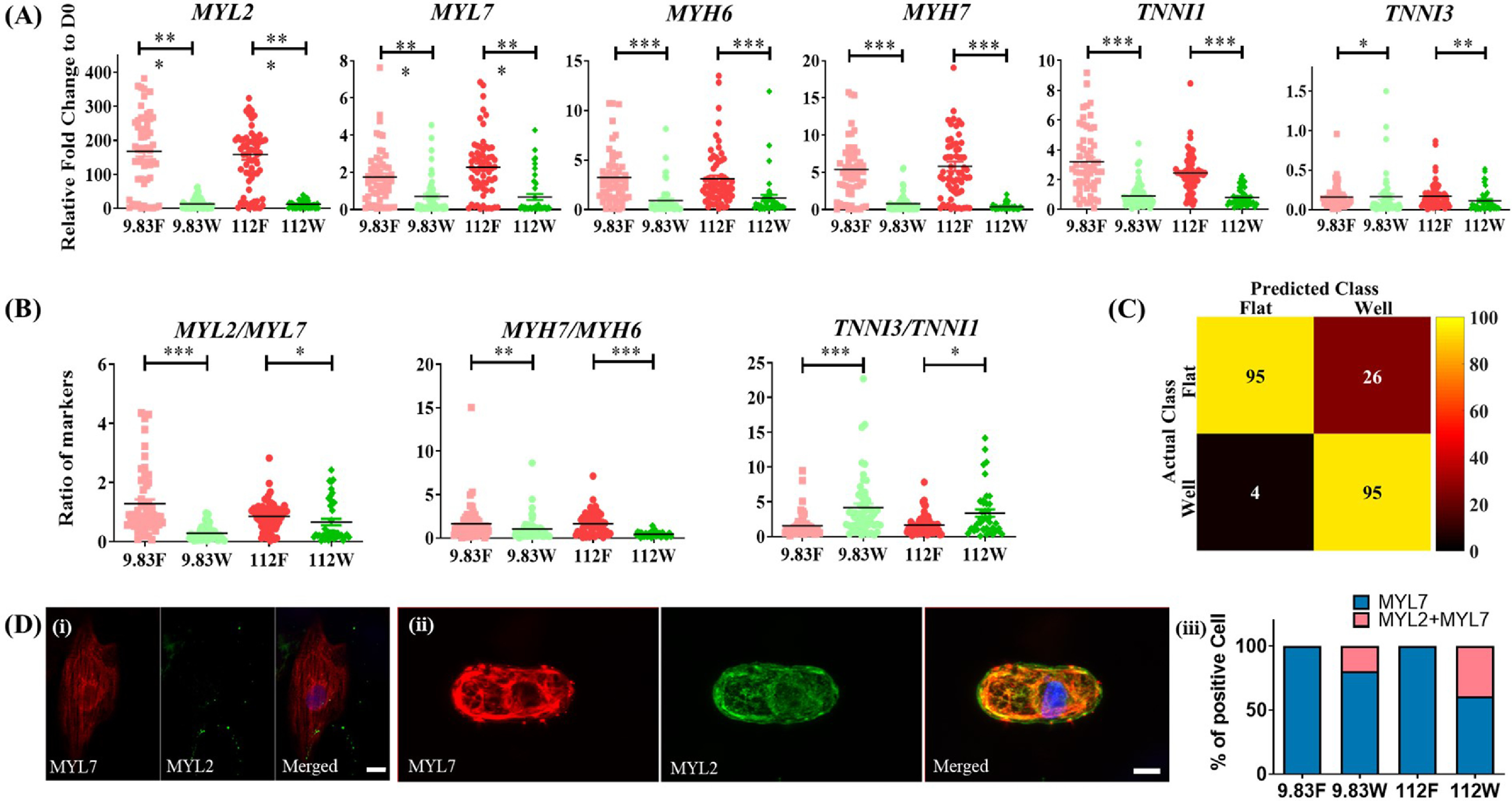
Multiplexing cell shape and substrate stiffness modulates gene and protein expression in isolated single hiPSC-CMs. (A) Single cell RT-qPCR analysis of the expression levels of cardiac structural markers, showing upregulated levels of MYL2, MYL7, MYH6, MYH7, TNNI1 and downregulated expression of TNNI3 in control flat surfaces with different substrate stiffnesses (2-way ANOVA, *p < 0.05, **p < 0.01, ***p < 0.001). (B) Ratio of cardiac structural markers. Values were normalized to hiPSC-CMs day 0 before plating. (2-way ANOVA, *p < 0.05, **p < 0.01, ***p < 0.001). Data shown as mean ± SEM. *N* = 4, *n* ⩾ 42. (C) Confusion matrix heatmap using a generalized linear model (GLM) of predicted class versus actual class of individual single hiPSC-CMs in each condition (Influences of biophysical cues provide on gene expression). (D) Immunostaining of hiPSC-CMs for MYL2 and MYL7, cultured on (a) flat controls, (b) 3D microwells on the platform and (c) bar graph showing the proportion of MYL2 and co-expression of MYL2 and MYL7 cultured on different conditions. Scale bars: 10 *µ*m. (9.83 F = physiologic stiffness flat control, 9.83 W = physiologic stiffness 3D microwell, 112 F = pathologic stiffness flat control, 112 W = pathologic stiffness 3D microwell).

A panel of structural markers, namely MYL2, MYL7, MYH6, MYH7, TNNI1 and TNNI3, was chosen based on previous reports on human cardiac development and structural maturation for quantification using single cell RT-qPCR [48, 54–56]. The cardiac structural markers showed upregulation during culture in all conditions compared to day 0, influenced by the geometrical confinement of the 3D microwell regardless of substrate stiffness (figure 4(A)) on day 7. The ratio between titin isoforms (TNNI3/TNNI1), known as a robust maturation marker, showed a four-fold and three-fold upregulation (physiologic and pathologic stiffnesses, respectively) in the 3D microwells of the platform as compared to their respective stiffnesses in flat control surfaces (figure 4(B)) on day 7, while MYL2/MYL7 and MYH7/MYH6 showed an opposite trend to TNNI3/TNNI1 with downregulation in isolated single hiPSC-CMs cultured in 3D microwells of the platform, compared to their respective stiffnesses in flat control surfaces. These changes demonstrated a differential effect on the myosin light chain sub-population related ratio (MYL2/MYL7) and a change in the hypertrophic myosin heavy chain ratio (MYH7/MYH6). Multivariate analysis was used to evaluate the influence of 3D microwells and substrate stiffnesses on this panel of genetic structural markers. Using a five-fold cross-validation multivariate generalized linear model (GLM), a model was created to discriminate CMs based on culture platform using genetic markers of a randomized selection of isolated single hiPSC-CMs (figure 4(C) (GLM—figure S3)). The model was then used to predict the substrate stiffness that the remainder of isolated single hiPSC-CMs most resembled based upon the measured signature of their gene expression. The model correctly assigns the geometrical culture condition, with 78.5% accuracy for predicting that isolated single hiPSC-CMs were cultured on a flat control surface and 95.9% accuracy for cells inside the 3D microwells. Thus, the analysis confirmed that the selected genes were differentially expressed between isolated single hiPSC-CMs residing inside the 3D microwells and on flat control surfaces of the platform, independent of substrate stiffness, emphasizing the effect of microscopic 3D shape on gene expression. Given the demonstrated strong influence of the shape constraint of the platform on gene expression, we hypothesized that protein transcription would also be modulated. MYL2 and MYL7 have been shown to be preferentially associated with atrial (MYL7) and ventricular (MYL2 and MYL7) myocytes. The protein expression distribution of MYL2 and MYL7 was analyzed with immunostaining, as shown in figure 4(D). As for connexins, the presence of 3D micropatterns and differential stiffness strongly affected MYL2 and MYL7 expression, as quantified in figure 4(D) (iii). The results show that isolated single hiPSC-CMs on flat control surfaces of both stiffnesses only expressed MYL7. However, co-expression of MYL2 and MYL7 was observed in isolated single hiPSC-CMs residing in the 3D microwells. The proportion of this co-expression was quantified to be higher on pathologic stiffness (112 kPa) as compared to physiologic stiffness (9.83 kPa) as shown in figure S4. An increase in MYL2 expression has been previously reported as a direct correlation of ventricular-like CM [53, 57, 58], but additional specific assays would be needed to comprehensively confirm a presence of this subtype. Therefore, isolated single hiPSC-CM confinement in the platform significantly affects individual cardiac structural gene expression with higher TNNI3/TNNI1-maturation ratio in isolated single hiPSC-CMs residing inside the 3D microwells, differential modulation of subtype population related gene-ratio (MYL2/MYL7) and protein expression of MYL2 and MYL7 with both microscopic 3D shape and stiffness.

### 3.5. Substrate stiffness controls calcium handling

**Figure 5.**
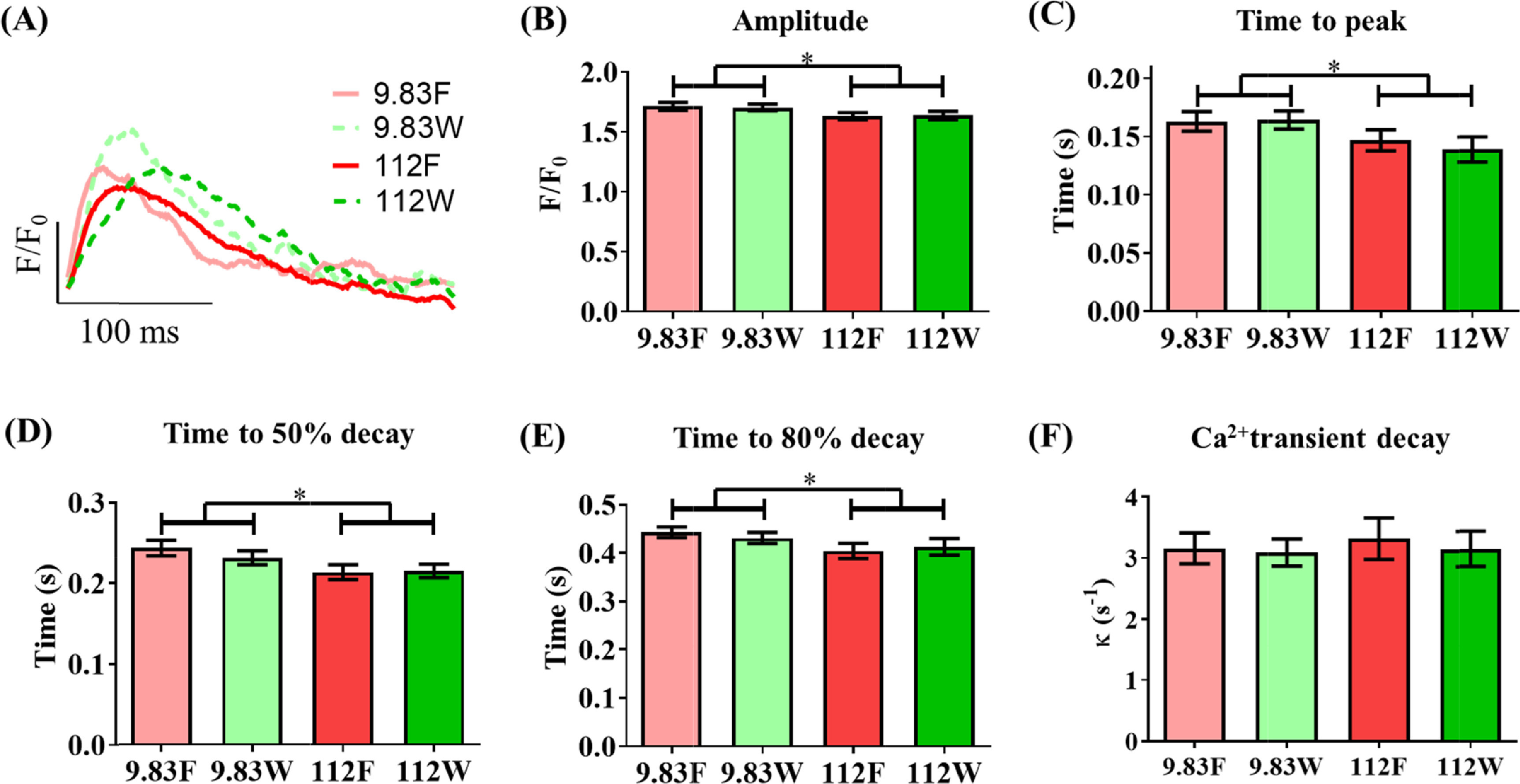
Substrate stiffness dominates influence on Ca^2+^ handling of isolated single hiPSC-CMs over cell shape. Ca^2+^ handling was assessed by using an isogenic hiPSC line harboring a genetically encoded calcium indicator, GCaMP6f, and optical mapping under field stimulation at 1 Hz. (A) Representative trace of intracellular Ca^2+^. Ca^2+^ transient parameters: (B) amplitude, (C) time to peak, (D) time to 50% decay, (E) time to 80% decay and (F) Ca^2+^ Transient decay. Data shown as mean ± SEM. *N* = 4 independent experiments, *n* ⩾ 36 (2-way ANOVA, *p < 0.05). 9.83 F = physiologic stiffness flat control, 9.83 W = physiologic stiffness 3D microwell, 112 F = pathologic stiffness flat control, 112 W = pathologic stiffness 3D microwell.

Finally, in order to investigate the function of isolated single hiPSC-CMs cultured in 3D microwells and on flat control surfaces of the platform, calcium handling was characterized using optical mapping with a GCamP6f-transgenic hiPSC-line (Movie S3). Fluorescence profiles of isolated single hiPSC-CMs under electrical field stimulation at 1 Hz were recorded using a Nikon inverted microscope equipped with a CMOS RedShirt camera. The data analysis was performed by a blinded investigator. Representative traces are shown in figure 5(A). The calcium magnitude of isolated single hiPSC-CMs cultured on pathologic stiffness was significantly reduced when compared to those cultured on physiologic stiffness of the platform (9.83 kPa) (figure 5(B)). Additional analysis revealed that the pathologic stiffness substrate (112 kPa) significantly abbreviated calcium kinetics (time to peak, time to 50% decay and time to 80% decay, figures 5(C)–(E), respectively). The 3D microwells of both physiologic and pathologic substrate stiffness did not affect the calcium cycling of isolated single hiPSC-CMs compared to those cultured on respective flat control surfaces. The rate of Ca^2+^ decay showed no significant difference between isolated single hiPSC-CMs when cultured on the flat control surfaces as compared to 3D microwells (figure 5(F)). This showed that substrate stiffness, but not 3D micropatterns, affected the change in calcium handling of isolated single hiPSC-CMs.

## 4. Discussion

Engineering surface topography to provide a niche that mimics the native context of cells is being actively exploited for biomedical research, including tissue engineering using single and/or multiple cell types [59]. Previously, entrapment of single cells and/or cell clusters with 3D micropatterns was piloted to be used in cancer research [60] and drug screening [61] with very limited use in the cardiac field. Previous studies showed the relationship between biophysical cues and CMs in many settings. Kevin Kit Parker’s group demonstrated that dynamic sarcomere configuration and force development can be observed using 2D geometrical cues [8, 62–64]. The effects of substrate stiffness on isolated single CMs showed that around 11 kPa CMs exhibited the highest contractility and calcium functionality [23, 65]. McCain *et al* and Ribeiro *et al* later studied the effect of substrate stiffness on CMs forced to acquire a specific 2D configuration. Single rat CMs showed biphasic responses to stiffness of cardiac structural changes and contractile work [7, 19]. These studies only assessed two dimensions. Two cell microtissues created by culturing two CMs, specifically a primary CM and a mouse iPSC-CM, showed correlation between functions and the amount of contractile force transmission between the cells [66]. However, these studies were conducted using rodent cells which might not be translated to a human setting and the studies have not dissected the mechanism. We previously created a 3D microgroove substrate that significantly influenced Ca^2+^ cycling properties of hiPSC-CMs and directionality in electrical signal conduction [67, 68]. This demonstrated the role of biophysical cues (i.e. cell alignment) on the functions of hiPSC-CMs with cell–cell contact influences. Wheelwright *et al.* studied functional maturation (e.g. size, dimensions and force production) on 2D micropatterning hiPSC-CMs by manipulating culture duration and extracellular calcium concentration on the functional maturation [13]. Our study is thus complementary to that of Wheelwright *et al*, and together they build a picture for how biophysical cues such as stiffness and shape together with culture duration and context might impact function of hiPSC-CM. A recent study from Abadi *et al* demonstrated the fabrication of a platform using adult CMs as a mold to form cardiac fibers [18]. However, this platform is limited to substrate stiffnesses similar to pathologic cardiac settings. Obtaining information on how a CM is influenced autonomously by its biophysical environment requires being able to maintain it in isolation, and physical cell–cell communication would restrict this. CMs are already known to display a strong dependence on substrate stiffness [19, 69, 70]. In this cell type, the mechanical environment alone can modulate contractility, tensile stiffness, sarcomere alignment, myocyte size and aging [71]. A biphasic correlation between substrate stiffness and contraction stress has been demonstrated, with the greatest force generated at physiologic stiffness (10–20 kPa in CMs) and governed by the Frank-Starling law [72]. However, in spite of the wealth of experimental evidence regarding the influence of topography and stiffness separately on hiPSC-CMs, their conjunction in an experimental system to assess the combined influence has not yet been demonstrated [73, 74]. In this study, we fabricated a platform to do this and applied multiplexed stimuli to isolated single hiPSC-CMs to address this question. Further refinement of the technology on the fabrication and biology sides could, for example, see the generation of chips providing on demand stiffness and geometry depending on the desired tissue or application. This can be extended to study ligand density, soluble factors and controlled number of cell-cell interactions. To build on key discoveries revealed in this study, we can build-in additional variables such as those listed above. In this way, we can more gain a wholistic understanding of how the extracellular niche impacts functions of hiPSC-CMs.

Importantly, we would not have been able to develop such a robust platform without a consistent and reproducible method of tuning substrate stiffness while permitting cell-ECM binding. PAAm has been a substrate of choice for *in vitro* work due to its ease of tuning, the reproducibility of the resulting stiffness over a large surface area and its low price [75], but conventional coupling of adhesion proteins by the photoactivatable Sulfo-SANPAH-mediated crosslinking reaction can be inconsistent, yielding a nonuniform protein topography. This has been demonstrated by Damljanovic *et al* [76]. To overcome this concern, we modified PAAm to improve the reproducibility and consistency of protein conjugation. Specifically, acrylic acid added carboxylic groups to the PAAm backbone that could be covalently linked with an amine group in proteins via EDC-NHS. The concentration of acrylic acid was tuned to obtain a uniform and highly robust protein conjugation without compromising stiffness reproducibility. This simple coupling method overcomes the frequently observed non-uniform coating due to variability in UV irradiation of photoactivatable cross-linkers [76, 77]. The result is a potent platform with potential applications that could be extended to other cell types such as fibroblasts, skeletal muscle cells, chondrocytes or adipocytes amongst others, where there is also a strong relationship between the shape and stiffness of the surrounding ECM and cellular function.

We chose to validate the capabilities of our system on hiPSC-CMs, given their exquisite sensitivity to the mechanical environment. Although hiPSC-CMs are intrinsically immature [78], with less organized sarcomeres, softer membrane stiffness [21], shorter calcium kinetics with lower amplitude, and differential expression of cardiac-genes [79] compared to human adult CMs, they have an inherent capacity to progress toward specific and more differentiated phenotypes, making them a suitable target to interrogate both basic and applied biomedical questions [56]. Applying our platform to investigate the effects of stiffness and 3D micropatterning on isolated single hiPSC-CMs can therefore answer two specific biological issues [1]: whether we can use this unique system to combine both stimuli to modulate cell phenotype, and [2] whether hiPSC-CMs are able to respond to these signals without the existence of physical intercellular communication (e.g. when they are in isolation).

In this study, we show that the 3D micropatterns of our platform are able to constrain hiPSC-CMs to adopt the dimensions of an adult myocyte, contributing to directing the cytoskeleton, and therefore the cells, as shown by the change in anisotropy and sarcomere orientation. This had been previously observed in 2D [63, 66]. In physiologic conditions, around 20% of isolated single hiPSC-CMs display a 60-degree difference which was not observed in the stiffer substrate, thus pointing towards a link between cell polarization and substrate stiffness [80, 81]. Isolated single hiPSC-CMs within the 3D micropatterns displayed a closer match to adult unloaded CMs in sarcomere length, subcellular localization and connexin expression (presence of Cx-43/Cx-40 and absence of Cx-45, both characteristics of adult working CMs) [53]. However, the functionality of these connexins needs further validation [82]. The presence of Cx-40 together with expression of only MYL7 in isolated single hiPSC-CMs on flat controls might indicate a higher percentage of atrial-like CMs although a further comprehensive analysis is needed [53]. In our work, we found a decrease in sarcomere length with an increase in stiffness with no significant difference in directionality. We have reported a sarcomere length of 1.62 *µ*m which might generate lower force than those hiPSC-CMs cultured on physiological stiffness that showed a sarcomere length of 1.77 ± 0.12 *µ*m, the optimal value found in unloaded CM [65, 66] and only 1.62 ± 0.05 um on pathologic stiffness. Rodriguez *et al* reported that sarcomere length increases with stiffness. However, the range of substrate stiffness is narrow and only ranges from 3 to 15 kPa [67]. This does not cover the range reported in our study. Jacot *et al* showed that neonatal rat ventricular CM generated greater force at the stiffness similar to native myocardium (10 kPa) than stiffer substrate stiffness. This could be speculated from suboptimal sarcomere length either higher or lower than the optimal point [11]. The decrease in sarcomere length found in pathological stiffness in our study is in line with Jacot *et al*, and directly indicates that the sarcomere length is suboptimal. Recently, Ribeiro *et al* and others showed a decrease of relaxed sarcomere length with an increase in stiffness using a novel alpha-actinin reporter. The study showed that the length decreased from around 1.8 *µ*m to 1.75 *µ*m when the stiffness increased from 4 kPa to 21 kPa and higher [18, 68]. The setup is very similar to our study in that the hiPSC-CMs were cultured as a single cell in a 3D micropattern. Although Ribeiro *et al* did not modulate stiffness like we do, their study demonstrated a similar inverse relationship between sarcomere length and stiffness as has been found in our study. The key determinant of the Frank–Starling relationship in CMs is the sarcomere length–tension relationship. As the resting sarcomere *α*-actinin length increases, more cross-bridge cycling occurs when muscles are stimulated to contract. The resulting tension increases and maximum force is produced when sarcomeres are within the optimal resting length [56]. Therefore, although it has not been measured in our study, this recapitulation of physiologic sarcomere length observed in the platform may well result in higher force production [13, 83]. In addition to the structural benefit imparted by the 3D microwells, the membrane stiffness of isolated single hiPSC-CMs cultured on the 3D microwell of the platform was shown to be higher as compared to its peer flat controls, in line with the reported increase over time taking place during cardiac development (9.83 kPa) [49, 84].

Single cell gene expression analysis revealed the significant influence of the physical extracellular milieu of our 3D microwells of the platform. An increase of MYL2 reported in this study on the stiffer substrate has been previously reported, probably stemming from the rigidity-sensing capacity of the myosin isoform [20]. Downregulation of MYH7 was also observed in this study. Many studies have shown that its upregulation is one feature of maturation and aging [85], but MYH7 has been also closely linked to cardiomyopathy [86, 87]. Additionally, the TNNI3/TNNI1 and MYL2/MYL7 ratios were significantly lower and higher, respectively, in isolated single hiPSC-CMs cultured on flat control surfaces for both pathologic and physiologic conditions than in 3D microwells, indicating the capacity of 3D microwells in the platform to modulate CM maturity and phenotype, respectively [48, 49, 58, 88, 89]. The close link of the MYH7/MYH6 ratio with the development of hypertrophy provides proof of an extra dimension that can be modulated by our platform, demonstrating the capability of the extracellular 3D environment to model a diseased state [90]. Moreover, we show for the first time that shape-confinement of isolated single hiPSC-CMs in 3D microwells on our platform can influence the differential expression of markers involved in CM identity, MYL2, MYL7, Cx-40, Cx-43 and Cx-45, pointing toward the ability to direct CM phenotype solely by applying the desired physical stimuli, decoupled from any physical cell-cell interactions. Further protein expression assays can be conducted to confirm protein expressions and their correlations [91]. The findings from proteins assays would provide an useful extra dimension to the present methodology regarding the biological induction of different populations of hiPSC-CMs [92]. Together, the increment in gene expression, the increased TNNI3/TNNI1 and cellular stiffness, and the decrease in the hypertrophic associated ratio MYH7/MYH6 of isolated single hiPSC-CMs residing inside 3D microwells compared to those cultured on flat controls, emphasizes the unique ability of the biofabricated platform to guide cell phenotype by manipulation of the multiple extracellular physical cues. This shows the increased degree of gene maturity and 3D microwell capacity to manipulate myocyte phenotype (MYL2, MYL7, Cx-40, Cx-43 and Cx-45 expression) and demonstrates that the microscopic 3D system strongly directs gene expression on isolated single hiPSC-CMs regardless of substrate stiffness. Future iterations will be focused on exploiting this unique capacity.

Of note, we found no substantial effect of single-cell 3D microwell confinement on calcium cycling, while substrate stiffness was found to be an influential factor. Isolated single hiPSC-CMs on physiologic stiffness (9.83 kPa) displayed increased amplitude and prolonged calcium transients due to an increase in time to peak and time to decay, compared to those cultured on pathologic substrate stiffness, as previously reported [17, 22, 69, 93, 94]. This is due to the rate of decay being unchanged. Therefore, the duration of the transient is only mirroring the lower amplitude. These differences are similar to the response of adult cardiac tissue in disease, whereby an increase in stiffness due to fibrotic remodeling (>50 kPa) causes the inversion of the force-frequency relationship in calcium cycling. This subsequently triggers an increase in heart rate to balance the decrease in ejection fraction as a decompensation mechanism. The mechanism requires an abbreviation of calcium transients, as we found in isolated single hiPSC-CMs cultured on pathologic substrate stiffness. However, it is possible that the induction of changes by the confinement in the 3D microwell of our platform requires longer than the 7 d period employed in this study, in order for the cell to develop all the adequate calcium cycling machinery, including well-structured T-tubules and more mature mitochondria. Finally, cell-cell communication could still occur from the chemical paracrine route.

Our platform enables simultaneous application of stimuli in a highly controllable manner, providing an easy approach to manipulate isolated single hiPSC-CMs, or any other cell type, and a tractable system in which to study *in vitro* cell development. This allows researchers to study a single entrapped cell, as well as potentially to study the effects of two or multiple cells by changing the size of the 3D microwell in our highly controlled experimental platform.

## 5. Conclusion

We microfabricated an improved platform to interrogate the effect of multiple biophysical cues on isolated single hiPSC-CMs. Specifically, we developed a novel and highly tunable platform capable of concomitantly delivering 3D microscopic adult-like CM topographical and stiffness cues to isolated single CMs, without any confounding influence of physical cell-cell communication. Our results show that this system can modulate calcium handling, gene expression and structural organization of isolated single hiPSC-CMs. This indicates that by solely manipulating the physical properties of the extracellular substrate, isolated single hiPSC-CMs can be directed towards a differential phenotype. Although CMs do not naturally exist in isolation, our system provides a tool to decouple the influence of physical cell-cell communication, focusing solely on physical cues, and demonstrates the high plasticity and adaptation to their microenvironment. In addition, this platform broadens the possibility to produce hiPSC-CMs with a known phenotype that is tailored for specific applications. Together, these results shed light on how fundamental developmental processes are regulated via multiple physical cues at the single cell level. We expect that this system will be applicable to other cell types where maturity and shape are similarly closely related and subsequently translated to multicellular *in vitro* models for developmental and clinical studies [5].

## Data and materials availability

Raw data is available upon request from rdm-enquiries@imperial.ac.uk.
